# Internet of Robotic Things in Smart Domains: Applications and Challenges

**DOI:** 10.3390/s20123355

**Published:** 2020-06-12

**Authors:** Laura Romeo, Antonio Petitti, Roberto Marani, Annalisa Milella

**Affiliations:** Institute of Intelligent Industrial Technologies and Systems for Advanced Manufacturing (STIIMA), National Research Council (CNR), 70126 Bari, Italy; antonio.petitti@stiima.cnr.it (A.P.); roberto.marani@stiima.cnr.it (R.M.); annalisa.milella@stiima.cnr.it (A.M.)

**Keywords:** internet of robotic things, industry 4.0, cyber-physical systems, remote working, agriculture, manufacturing, health-care, education, surveillance

## Abstract

With the advent of the Fourth Industrial Revolution, Internet of Things (IoT) and robotic systems are closely cooperating, reshaping their relations and managing to develop new-generation devices. Such disruptive technology corresponds to the backbone of the so-called Industry 4.0. The integration of robotic agents and IoT leads to the concept of the Internet of Robotic Things, in which innovation in digital systems is drawing new possibilities in both industrial and research fields, covering several domains such as manufacturing, agriculture, health, surveillance, and education, to name but a few. In this manuscript, the state-of-the-art of IoRT applications is outlined, aiming to mark their impact on several research fields, and focusing on the main open challenges of the integration of robotic technologies into smart spaces. IoRT technologies and applications are also discussed to underline their influence in everyday life, inducing the need for more research into remote and automated applications.

## 1. Introduction

Smart services are becoming increasingly fundamental with the growth of the fourth stage of industrialization, also addressed as Industry 4.0, where new disruptive technologies are changing both industrial and research fields [1]. During the first three industrial revolutions, there has been an improvement of productivity thanks to the creation of new mechanical, electrical, and electronic technologies. In the last few years, the need for an improvement in human life quality has led to realize more and more production models of personalized and digital services [2].

The main result of the present fourth industrial revolution lies in the development and rapid spreading of the Cyber-Physical Systems (CPSs). CPSs manage to develop applications merging physical assets to computational capabilities. CPS technologies cover a wide range of applications, such as electrical power grids, transportation systems, health-care devices, gas distribution, and so on. In CPS, the interaction with the physical systems occurs through networks, running complex analysis while extracting data.

The use of networking, internet, and sensors in CPS leads to the definition of Internet of Things (IoT) [3]. IoT can be seen as the infrastructure that makes CPSs possible, as IoT systems are based on communication protocols, thanks to which physical assets manage to connect with each other, transferring and exchanging information. While the IoT paradigm is not intended to analyze data and information systems, CPSs are meant to exploit IoT communication architecture to run complex analysis through a centralized analysis hub, where information can be extracted from raw data to send control commands to the physical asset.

**Figure 1:**
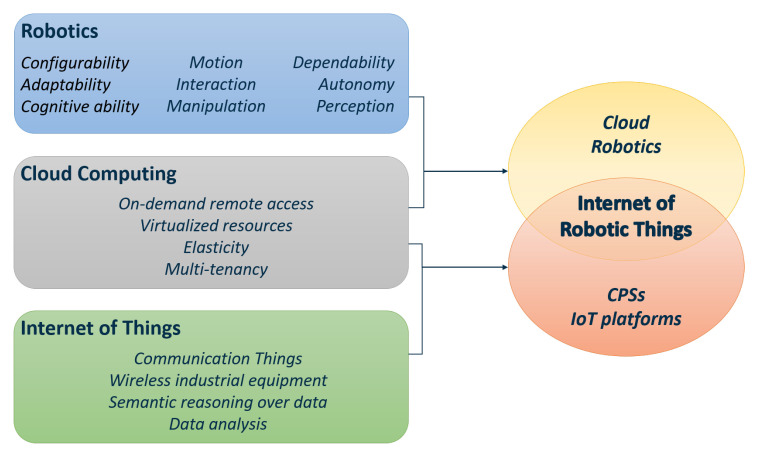
Highlights of Robotics, Cloud Computing and Internet of Things, which merge into the Internet of Robotic Things.

Both IoT and CPS lay a strong foundation for the development of a new research area: the Internet of Robotic Things (IoRT). This new concept has brought several changes in different domains that cover several applications [4], including systems that have to work in challenging environments. For instance, IoRT systems can be adopted by manufacturing industries to autonomously and remotely perform challenging tasks such as assembling, packaging, welding, managing quality control, and so on. Moreover, IoRT systems are also spreading outside the industry environment, in museums, sports, and entertainment [5]. Nevertheless, their development is mainly due to the necessity of creating interconnected systems in the context of Industry 4.0 [6,7], merging digital and physical worlds. In this scenario, IoRT represents the core of robotics-embedded IoT systems, where cloud computing and networking can be implemented to accomplish elaborated tasks, allowing robots to share, network, and gather different kinds of information among both humans and machines. A scheme of robotics, cloud computing, and IoT integration can be observed in Figure 1.

Many reviews have been written in the last few years [8,9,10,11,12], yet none of them classifies the IoRT systems according to smart domains. This survey is intended to shed light on the IoRT applications associated with different smart domains in the context of Industry 4.0, defining the most recent state-of-the-art of IoRT technologies and outlining how IoRT systems could play a key role in our society. The main goal is to outline the major challenges in each field, aiming to understand in which areas the implementation and study of IoRT applications must be further investigated. As this manuscript is mainly focused on the industrial and production fields, IoRT applications in smart manufacturing and smart agriculture are deeply analyzed. Further domains are then explored, such as health-care, education, and surveillance, to outline how IoRT systems are spreading in many aspects of everyday life.

The main contributions of this survey involve:

- a picture of the interaction between physical and virtual scenarios managed by the Cyber-Physical Systems technologies;
- the definition of the Internet of Robotic Things concept: its architecture and main technologies;
- the identification of various application domains, outlining the newest state-of-the-art literature based on IoRT technologies;
- open issues and challenges that are worth investigating in the future, showing how IoRT systems could represent a key role in the context of the fourth industrial revolution.

The manuscript is organized as follows. Section 2 illustrates the main features involved in Industry 4.0, focusing on technologies based on the CPS. Subsequently, an outline of the IoRT is provided, analyzing its components and its architecture. Section 3 defines the latest domains and applications where robotic systems are integrated with IoT technologies. Finally, Section 4 gives a discussion regarding open issues and challenges, while Section 5 draws the conclusions of the survey.

## 2. Smart Technologies in Industry 4.0

The fourth industrial revolution, commonly known as Industry 4.0, can be described as the next level of manufacturing, where digital integration and intelligent engineering are used to transform the way machines communicate with each other and humans. The growth of Industry 4.0 has led to an improvement of the entire manufacturing industry [13], giving birth to the so-called Smart Spaces [14] and, more specifically, Smart Factories. The main function of Smart Spaces is to monitor applications and processes, such as power consumption or state of sensors and actuators, in a defined controlling area. In particular, the term Smart Factory refers to a new approach to industrial manufacturing, where IoT technologies are used to control the manufacturing process, while gathering, analyzing, and exchanging information [15].

Strictly connected to the smart factory is the concept of Smart Machine [16]. These IoT-based machines include controllers, add-on sensors, and use real-time data from their own components and other machines to attain self-awareness and self-comparison [17]. Self-awareness enables machines to make diagnostics regarding possible malfunctioning components, while self-comparison enables the machine to configure their settings properly, based on their working history. For such purpose, network architectures and communication protocols, both wired and wireless, are used to coordinate sensors and actuators for more complex industrial applications. Industrial robots play a key role in the Smart Manufacturing environment, where operations such as assembling, welding, spray painting, and so on are merged with IoT technologies to guarantee interaction among multiple devices and robots, assuring reliable and efficient production [18].

### 2.1. Cyber-Physical Systems

One of the most prominent factors related to Industry 4.0 is the so-called Cyber-Physical System (CPS). With the continuous development of different technology domains, CPS applications have become fundamental, as they manage to connect all physical devices to the Internet, merging virtual and physical worlds to attain smart products and production [19]. Physical-world, cyber-space, and communication networks are the core of CPS structure: (i) physical world refers to those physical objects, processes, and environments to be monitored or controlled, (ii) cyber-space represents those information systems such as services, applications, and decision-making units, while (iii) communication networks refer to those components that manage to link the cyber-space with the physical-world.

The application of CPS in production scenarios, where machines, sensors, and actuators are linked to attain the highest efficiency in terms of production, leads to the concept of Cyber-Physical Production System (CPPS). CPPS lays its foundation in the context of the so-called Digital Twin [20], where physical modules are linked to virtual modules, aiming to create a connection between physical elements and the corresponding digital version [21]. With the advent of the fourth industrial revolution, control and production systems have gained much importance in many smart domains. Particularly, smart products and smart production systems are strictly related to the CPPS, and different architectures have been designed to deal with failures in discrete-event processes [22].

CPPS can be helpful in improving the flexibility of IoRT-based production systems in smart domains, such as manufacturing, agriculture, medical surgery, and elder care. Specifically, Human–Robot Interaction (HRI), where humans and robots work together, can be applied in these fields just mentioned, particularly in manufacturing. Here, the information given by the physical contact between humans and robots can be used in production systems such as assembling and welding. In such contexts, defining an HRI architecture is fundamental in the design and implementation of CPPS [23], aiming to affect the trajectory of a robot based on points given by human workers.

**Figure 2:**
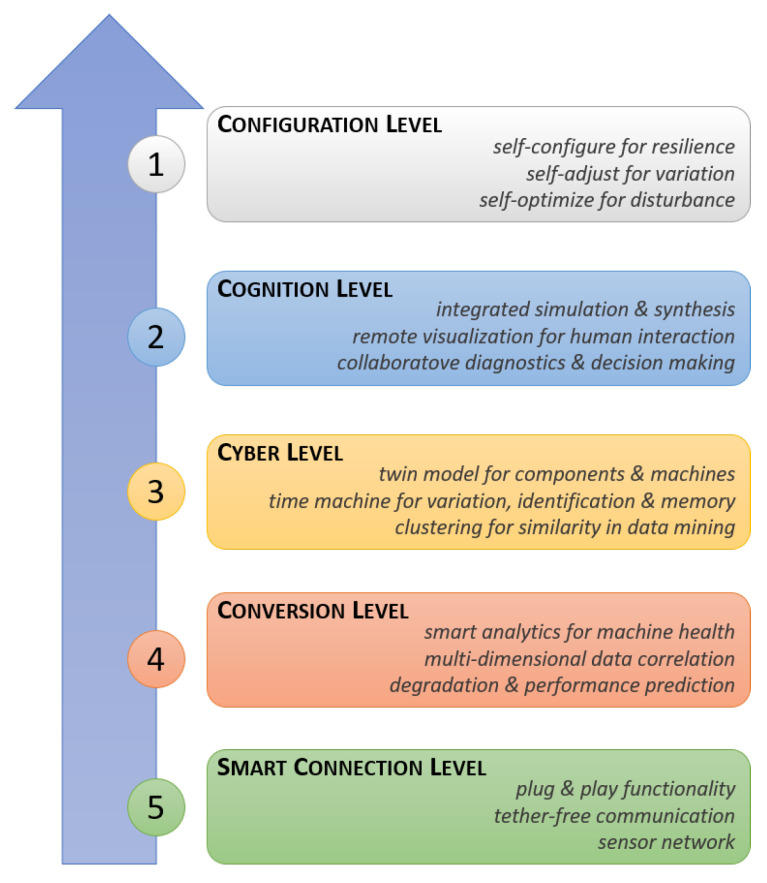
Five-layer architecture of CPS implemented in industrial settings. Lower levels collect data, which is analyzed and condensed at each upper level. With this configuration, information fed to each higher level is more valuable than information coming into the level below.

As CPS is the first stage of development, it reveals to be fundamental to define its structure and methodology. In industrial settings, the architecture of CPS is characterized by five different levels [24], which can be observed in Figure 2. Such structure, known as 5C architecture, manages to clarify how to construct a CPS from the initial data acquisition to the final value creation. Nevertheless, several CPS architectures have been developed, each focusing on different aspect, which aim to better characterize industrial systems and smart factories in the context of Industry 4.0 [25,26]. In the following, the levels of 5C architecture are outlined.

The *Smart Connection* level deals with the acquisition of accurate and reliable data, which can be directly extracted from proper sensors or manufacturing systems. Such data are transferred to the central server using specific protocols, where the *Conversion* level manages to extract meaningful information by means of algorithms and methodologies that are developed with respect to the application under consideration. In the *Cyber* level, a massive amount of data are gathered. Here, additional information is extracted to provide better insight on the status of each machine among the system, aiming to give machines self-comparison ability. Such information is sent through proper graphics in the *Cognition* level, where knowledge about the machine status can be acquired, aiming to compare information and make the correct decision to optimize the maintaining process. Finally, the feedback from the cyber-space to the physical space occurs in the *Configuration* level, acting as supervisory control to make machines self-configurable and self-adaptive.

The growing need to use sensors and networked machines leads to the development of a large cluster of information, known as Big Data [27]. CPSs can be useful to manage both Big Data and the interconnection among machines, making the factories suitable for the Industry 4.0 era. Generally, CPSs consist of two main features: (i) the capacity of acquiring data in real-time from the physical-world, and an advanced connectivity that manages to gain information feedback from the cyber-space; (ii) computational power, intelligent data management and analysis, which are integrated into the cyber-space. Therefore, CPSs are able to realize a dynamic collaboration with physical systems. The latter collects data by distributed field devices in CPSs, which aims to guarantee the real-time capability and accuracy of the collected data. CPSs have a wide range of applications, such as industrial control [28], distributed energy system [29], digital medical field [30], etc.

### 2.2. Internet of Robotic Things

**Figure 3:**
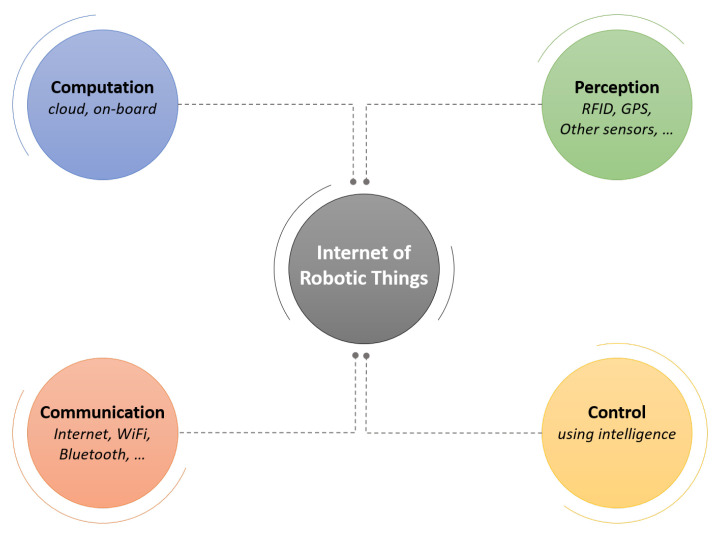
Overview of IoRT components.

In recent years, the fourth industrial revolution has led to the development of the so-called Internet of Robotic Things [9], which manages to attain systems with decision-making autonomy, perception, and manipulation. An outline of the IoRT components can be seen in Figure 3.

The most advanced concept in terms of robotics lies in IoRT, where CPSs are used to lay a strong foundation in enhancing IoT itself [10]. In IoRT systems, modern robotic technologies have been merged with cloud computing [31] and networking, integrating CPSs and IoT protocols to develop new technologies. As an outcome of such integration, smart devices become able to monitor events, merge sensor data from a variety of sources, and use local and distributed intelligence to determine the best course of action [32]. This new approach features different technologies to perform complicated tasks and operate in a heterogeneous environment [8]. Furthermore, Wireless Sensor Networks (WSN) are becoming a matter of considerable importance within the latest years. WSNs are widely investigated in the literature [33], considering how many new challenges they imply, and how many new methodologies have been deepened to address them. Specifically, the necessity of deploying sensor nodes according to certain algorithms and situations leads to the possibility of integrating such sensors with robot networks [34]. Such robots cohabit with sensors, cooperating to enhance the potential of the WSN. Consequently, the implementation of robot networks with wireless sensors has contributed to the development of new IoRT-based technologies and applications that can address different tasks, from deployment to communications.

Robotic systems have brought considerable changes in various aspects of human life. Both in the industrial and the academic worlds, robots have been used in performing all sorts of complex and challenging tasks such as packaging [35], assembling [36], welding [37], etc. In this context, IoT and robotics convergence occurs in the development of new heterogeneous robotic systems, with the aim of improving the autonomous behavior of the robots. Moreover, the integration between robotics and networking is of critical importance in the development of the IoRT systems. Networked Robotics occurs to merge robot system architectures (both hardware and software) and applications that use networks, such as the Internet and cloud computing [38]. In recent years, the evolution of the Internet and robotics has led to networked robots spreading in many fields, becoming fundamental in the integration of robots, cloud computing, databases, and even humans all over the world, as they can be based on a Local Area Network (LAN), or distributed over a Wide Area Network (WAN). IoRT systems exploit such technology, managing to integrate robots with smart sensors that can operate in the network and exchange information. Formally, the term IoRT refers to an ecosystem of intelligent devices able to monitor events, gather and analyze data from different sources, exchange information, use local and distributed intelligence to determine an optimal sequence of actions, and then act to change the physical environment while physically moving through that environment.

**Figure 4:**
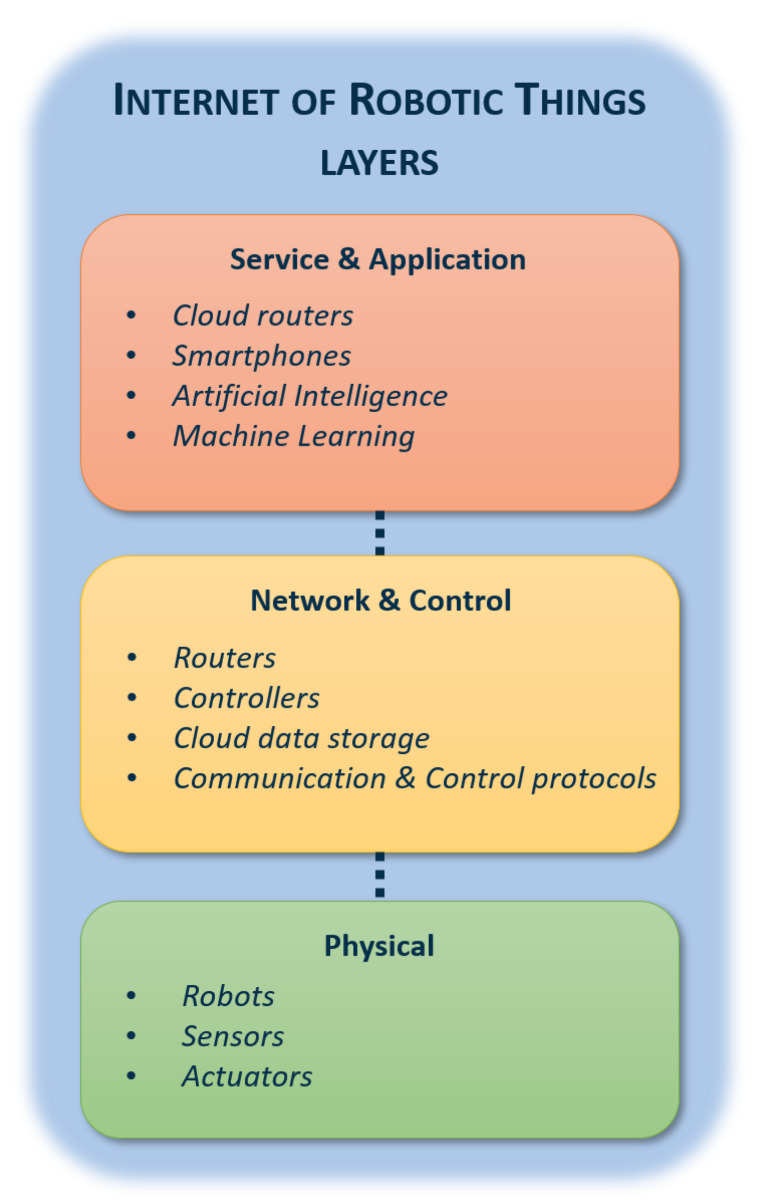
Schematic diagram of Internet of Robotic Things system architecture.

The key architecture of the IoRT systems, as depicted in Figure 4, consists of three main layers [12]: (i) Physical layer, (ii) Network and Control layer, and (iii) Service and Application layer.

The Physical layer represents the lowest level of the IoRT architecture, which includes robots, sensors, and actuators. Generally speaking, the word robot refers to vehicle, drones, unmanned vessels, and so on. The words sensor and actuator, on the other hand, refer to any kind of system used to perceive and act in the environment, respectively, considering a wide set of systems ranging from home appliances to industrial sensors. Robots cooperate to develop multi-robot systems, which integrates multiple agents operating in the same environment. Such technology can develop and upgrade smart applications regarding robotic systems, remotely manage distributed activities, and increase fault tolerance, to attain an improvement of the overall system performances [39]. Furthermore, sensors and actuators can be integrated into robot applications, aiming to optimize, monitor, and control various processes such as navigation, calibration, and tuning [40].

Nevertheless, complete and efficient integration of sensors and actuators with robot applications occurs in the Network and Control layer, where different components can rely on certain protocols to communicate and control processes. The second layer of the IoRT architecture includes controllers, routers, servers, and various communication and control protocols. In particular, communication techniques such as WiFi, 6LoWPAN [41], Bluetooth Low Energy (BLE), Radio Frequency Identification (RFID) [42], Near Field Communication (NFC), and WSN based communication [43] are used to attain smooth information transmissions among robotic systems of both short and long distances.

Finally, the Service and Application layer represents the top level of the IoRT architecture, which includes the implementation of programs in order to control, process, and analyze both environmental parameters and agents (robots, sensors, and actuators) in smart environments. In addition, this layer includes Artificial Intelligence and Machine Learning algorithms [44], which can guarantee smooth integration among robotic systems and IoT applications, to attain optimized solutions for complex problems in physical environments.

## 3. Smart Domains and Applications in the IoRT Systems

With the fourth industrial revolution, where robotics, CPS, and cloud technologies are merged, different domains are benefiting from rapid development [45]. In this context, the IoRT systems can provide several advantages over traditional robotic applications, such as offloading computation-intensive tasks on the cloud, accessing large amounts of data, and sharing information with other robots, aiming to learn new skills and knowledge from each other [46]. Moreover, IoRT applications can also be used remotely, facilitating the work of both researchers and industrial operators, and making it more accessible, allowing cooperation between humans even from long distances.

In the following, different domains are defined, in which IoRT solutions have been approached and implemented. Table 1, Table 2, Table 3 in the following subsections outline the scenarios covered in this section, highlighting the varieties of the latest technology addressed in different environments. However, the gathered literature covers different domains, and it is worth noticing that the methodological approach is often similar, as well as the technology used.

### 3.1. Manufacturing

Robot-based production represents the backbone of smart manufacturing, and the concept of industrial robots has been occurring a continuous change in the latest years, mainly due to the embedding of IoT technologies [47]. IoRT represents the main enabler of such change, through which manufacturing is embracing the concepts of Industry 4.0, embedding sensors, automation, and monitoring of products and processes [48]. The Fourth Industrial Revolution has transformed how products are manufactured, adapting to such technological innovation, aiming to produce high-quality goods and services. Table 1 shows an overview of the literature gathered, related to smart manufacturing.

Smart manufacturing involves system flexibility, monitoring, and adaptation to change. Specifically, additive manufacturing is a critical process in terms of manufacturing methods. In fact, innovations in digital technologies that are occurring during the Fourth Industrial Revolution need to keep up with advancements in manufacturing processes and materials [49]. In this scenario, with the aim of facilitating smart manufacturing by sensor systems, flexible electronics of additive manufacturing and their reliability during processes are of critical consideration.

IoRT may include several applications in the manufacturing domain, such as spot welding and spray painting [60]. Specifically, spot welding refers to apply a welding tool to a certain object, such as a car body, at specified discrete locations [61], while spray painting involves covering a surface with an even coat of paint, pre-specifying the trajectory along which the robot will move [62]. Both applications may require real-time correction of the path to accommodate small deviations from the expected path, aiming to attain adequate production and avoid issues such as inaccuracy and collisions with both humans and robots.

As the ability to perform self-localization and navigating are of crucial importance for several industrial robots, path planning is currently a research challenge in the manufacturing domain [51,55,56], and different algorithms are being studied to resolve this issue, such as Bellman–Ford [63], Dijkstra [64], and Floyd Warshall [65] algorithms. Furthermore, industrial manipulators provided with visual guidance can further enhance path planning systems, as a vision-based approach gives more awareness to detect and avoid obstacles [50,58].

The main concern regarding path planning is the negative impact on the computational speed of the path algorithm that can be caused by large data storage and complex computing processes. Therefore, it is necessary to improve the efficiency of the path planning algorithm [66], as well as include a cloud computing approach, aiming to manage hardware resource constraints of industrial robots [67].

With cloud computing, task executions are established invoking cloud technologies, assuring integration and interoperability [68]. In manufacturing, the assembly process has also changed in the Industry 4.0 era, evolving into the robotic assembly line, where robots manage to perform tasks previously performed by human labor [69]. The assembly process corresponds to mate different components with the aim of developing a new sub-component, or a finished product. IoRT technologies can enhance such process, taking advantage of embedded CPSs [52,53], aiming to use multiple robots to allow components to be re-positioned or manipulated, executing more complex assembly procedures.

Moreover, the introduction and development of cloud robotics have completely changed and improved the flexibility and extensibility of task scheduling in manufacturing [70]. By interacting with the cloud, industrial robots become able to download data regarding the manufacturing architecture and the other robots included in the system, managing to overcome several limitations about information and learning [71]. Therefore, the computational power of the cloud servers compensates for the limited computational power of the robotic system itself. As an example, [57] defines a novel cloud-assisted and self organized manufacturing system to integrate the manufacturing applications in the cloud paradigm. More specifically, the work is focused on a personalized candy packing application, interconnecting physical shop-floor entities, such as robots, conveyors and products, and client terminals. During the interaction process, plenty of data are collected into the cloud terminal, and application software is deployed to process such data.

Another fundamental aspect of smart manufacturing involves collaborative robots, which are revolutionizing the robotics market. Collaborative robots are safe and intuitive to use, managing to assist human operators, adapting to uncertainty [72]. To such purpose, the location of the human, along with the location of her/his limbs, must be known in real-time, while the robot itself moves. Inertial sensor-based methods and vision-based can be useful to detect human presence inside collaborative work-space [73]. In literature, several areas of research deal with human–robot interaction (HRI), aiming to develop robots suitable in a certain work environment [54,59], taking advantage of sensors for manufacturing situation awareness. The integration of such IoRT systems in smart manufacturing is intended to leverage the strengths of both robots and human operators, aiming to compensate for the limitations of one another [74].

### 3.2. Agriculture

Advanced technology in Industry 4.0 has brought a considerable innovation in agriculture. Labor-intensive tasks, such as the harvest of fresh fruits and vegetables, are experiencing a substantial change due to the integration of IoT-based systems in the agriculture domain [75]. Even though most farmers are still reliant on traditional techniques, in recent years, there has been significant interest in developing smart agricultural systems, focusing on issues such as cropping yield, weed control, and data gathering from fields [76]. In this context, IoRT systems can be designed and implemented to empower farms with technologies that integrate services, products, and knowledge in order to increase productivity, quality and profit, taking advantage of the collaboration between humans and smart systems [77]. Table 2, summarizes the literature taken into account for this section.

The particular vulnerability of agriculture to climate change leads to the primary smart agriculture concepts: Climate-Smart Agriculture (CSA) and Sustainable Intensification (SI) [87]. The main goal of CSA is to increase incomes and food security, while decreasing green house gas emissions [88]. As climate change has a significant and generally negative impact on agriculture, farmers, and farm service providers must respond effectively in the long term for such issues, as well as being able to manage the risk associated with increased climate variability. In this context, on-farm water storage and irrigation, as well as upgrades of farm enterprises themselves, are of critical consideration, as it is fundamental building agricultural systems able to enhance their own reliability through soil, water, and plant fertilizer management [89]. To this purpose, CSA systems based on the IoRT architecture allow accessing crop varieties that are more tolerant of heat, droughts, floods, and salinity, aiming to go far beyond the simple goal of intensifying agriculture [90].

CSA and SI are strictly connected with each other, as they both address climate change focusing on diversification, exploiting complementarities between crops. Since farmers are not going to adopt practices for climate change adaptation that may not yield improved returns on investments in the short term, SI occurs to address resource scarcity and environmental challenges, increasing harvesting and arable crops, aiming to find the best approach to maintain the trade-off between yields and environmental needs, even across different circumstances.

While in some areas yields are compatible with environmental improvements, in other scenarios yields reductions or land reallocation can be necessary in order to ensure sustainability and guarantee benefits such as wildlife conservation [91], carbon storage [92], flood protection [93], and so on. To this aim, data analysis gathered by means of IoT systems [94] and mobile robots, combined with cloud computing, can provide practical information regarding the level of water resources, humidity, chemicals, and so on [85]. In addition, image recognition and processing techniques, such as crop image acquisition, can further exploit such information. In the IoRT systems, crop image acquisition architecture, based on cloud computing and wireless network, can increase flexibility and adaptation of mobile vehicles [80,86], overcoming the traditional image acquisition systems which mainly depend only on a fixed camera or the mobile robot itself.

In agriculture, robotic systems can be decisively helpful in attaining both high quality and quantity products, as human skills and agricultural machinery are deeply limited with respect to robots expertise. The process of robotic integration in agricultural environments has led to the robotization of those machinery that plays a key role in agriculture, such as irrigation and fertilizer systems, harvesters and tractors [95]. The integration of IoRT systems in agricultural machinery requires a new approach to manage the control signals from the control system towards the actuators. Such systems should guarantee an improvement in the economic viability, reducing environmental impact and increasing food sustainability [75].

IoRT-based smart agriculture systems are designed to perform various agricultural activities, such as moisture sensing, irrigation, crop monitoring and defence against pests and harmful animals [96]. To this purpose, mobile robots with integrated Global Positioning System (GPS), such as areal and ground vehicles [79,81], are used to collect data from the field [97,98]. Unmanned Aerial Vehicles (UAVs) are often used along with a set of IoT devices, to gather information from such sensors, overflying the territory [99].

Precision agriculture occurs to use an IoRT approach to deploy herbicide, fertilizer, or irrigation, aiming to manage different scopes, such as variations of crop sizes, light, and weather conditions [78,82]. The main goal of this farm management approach is to efficiently reduce farm resources, limiting the cost of agricultural production, while maximizing the yield. To reach this goal, a network of intelligent sensor can be used to monitor and measure any change in plants, using a network of intelligent sensors [83,84]. In this context, the WSN system, composed of a limited number of nodes integrating radio frequency (RF) transceivers, micro-controllers, sensors, and power sources [100], can provide further support in gathering, controlling and monitoring of data [101,102]. IoT devices are deployed in a specific area, aiming to monitor environmental parameters [103], using a wireless connection to send data automatically via multi-hop communication. On the other hand, due to the limited autonomy of the devices, it is fundamental to deploy the node sensors heterogeneously, aiming to have the widest coverage with minimum energy expenditure [104].

WSN applications are useful in smart agriculture, as their main features include self-configuring, diagnosis, and self-organizing [105]. Such properties offer high spatial and temporal resolution to monitor crops through the sensor nodes deployed across the agricultural fields [106]. In [84], a sensor network is automatically deployed by a mobile robot, which is used to look for the best suitable position of the deploying node sensors, aiming to gather as much data as possible from the surrounding environment. A comparison to a manual deployment has been conducted, showing that the robotic-aided approach leads to higher performance, managing to increase communication efficiency, aiming to monitor environmental parameters in wide outdoor environments. The deployment of an IoT network in smart agriculture, aside from other benefits, can limit significantly the maintenance costs. Such agricultural systems, in fact, can provide data by means of sensors measuring heat, moisture, chemicals, etc. [107]. With such information, water, fertilizer, and pesticides can be autonomously deployed through a robotic system, in more precise quantities and positions, and with better time scheduling to increase yields and decrease costs.

### 3.3. Further Domains: Health-Care, Education, and Surveillance

IoRT systems are considered to have a huge value in health-care, education, and surveillance domains. Table 3 comprises an overview of the literature considered for this section. Specifically, IoRT can offer health, societal, and economic benefits in several applications, in particular for patients groups with certain needs, such as mental disability, stroke patients, sufferers, amputees, and so on [108,109]. The integration of robots with sensors and IoT devices gives several advantages in providing real-time health information and diagnosing patient conditions, aiming to decrease the risk of human mistakes such as diagnosing wrong drugs, doses, and procedures [110]. In addition, the IoRT technologies can bring many advantages in other applications such as tracking patients, staff and ambulance, automatic data gathering, and sensing [111].

Focusing on the education domain, robots require using appropriate and adaptive behaviors to attain and maintain adequate social interactions with people, aiming to become exploitable in assistance services such as homework and teaching [112]. Measuring the electrodermal activity (EDA) can be used for HRI, as it corresponds to a change in the skin conductance in response to episodes of attention, anxiety, and excitement [119]. Furthermore, EDA responses measured in children can differ considerably from the average responses measured in adults [120], as children may not respond to certain impulses as adults do. In such situations, the IoRT technologies used for child-educational concerns need to collect, process, and analyze data, in order to attain automatic prediction of children behavior and state.

The monitoring of places and people, with the aim of controlling human health and protecting certain environments, is a known concern. Here, IoRT systems play a key role, as they can provide smart technologies for high surveillance in environments such as sensitive areas, hospitals, military borders, public places, and homes [113,115]. Generally, closed circuit television (CCTV) cameras are used for supervision of both indoor and outdoor surroundings [121]. Nevertheless, such technology entails several issues and constraints, mainly caused by potential tampering and the presence of blind spots. Such drawbacks can be partially solved by increasing the number of cameras in the system [17], covering more nooks, yet increasing the cost and the complexity of the system itself.

The integration and development of the IoRT applications in surveillance appear to be the most suitable choice in surveillance contexts, as the IoRT systems can be programmed and implemented to be fast and work efficiently, covering larger areas in order to make secure a certain space [114]. In addition, cloud technologies integrated with robotics, which assure real-time detection, are mostly useful to remotely monitor environments [117] such as homes [122], industries [123], retail and wholesale stores, and, more generally, to detect human presence in different scenarios [124]. Sensor readings such as GPS, magnetic field, quality of air, and environmental values can also be helpful in the supervision of both indoor and outdoor scenarios [116,118], as the robot can transmit real-time data during a surveillance mission [125,126].

## 4. Issues and Challenges

Internet of Robotic Things represents a new concept that is intended to outline the merging of robotics technologies with IoT systems and cloud computing. As an outcome of this integration, the interaction between IoT, cloud computing, and robotics research fields is developing rapidly. Innovative technologies and applications are allowing for integrating smarter robot systems that will guarantee interoperability, real-time capability, and autonomous collaboration. Nevertheless, such IoRT applications are not enough developed to be completely transferred into industrial scenarios, as the main experiments proposed in the corresponding literature run within academic laboratories.

The need for cooperation among multiple robots, sharing spaces with humans, represents a key issue in smart environments. Multi-robot coordination is still facing several concerns regarding consensus networks, manage control, communication towards both infrastructures and other robots, and coordinated trajectory tracking [127]. A particular challenge also depends on the lack of support for heterogeneous robot configurations. In multi-robot operations, it is considerably difficult to integrate, configure, and coordinate the IoRT technologies from different manufacturers, which often use different systems. In the research domain, several enhancements have been reached in enabling certain features in multi-robot applications, but further improvements necessarily require the active involvement of the robot manufacturers themselves. Moreover, human–machine interfaces are becoming more and more relevant in several environments, such as hospitals, restaurants, and service areas. In HRI, smart robots are supposed to respond to well-established human gestures [128,129]. At the same time, corresponding data processing aims to reach the maximum autonomy and safety in HRI. However, there are still many constraints that hinder HRI development. New typologies of HRI have been studied in the latest years, such as eye-tracking [130], voice interaction [131], and biological recognition [132], but they still need to be investigated, as most of them have been analyzed only in research laboratories [133], and they are not widely used yet.

With the development and advancement of the CPSs and Industry 4.0, more and more industries are adopting remote working solutions for security reasons, where human operators cooperate with robots remotely, reprogramming, and controlling them from a safe environment. In such scenarios, HRI assumes a new and more important meaning, as the need for advances in technology and applications can be extremely helpful in allowing human operators to remotely lead-through industrial robots [134]. Advancements in IoRT technologies could be the answer for a new, better way to manage industrial operations. Lately, remote working is getting a lot of attention due to the major benefits that it brings. It has been shown that remote working leads to increased performance in terms of production, resulting from an improved work-life balance [135]. IoRT systems can further enhance such advancements, opening new possibilities related to remotely working with industrial robots. Furthermore, the same paradigm can be easily exported to any other application of this survey. For instance, in the case of education, students can experience more benefits from the adoption of social robots arranged in an IoRT scenario. Remote education could be extremely helpful for a lot of students, particularly the ones affected by diseases that force them to stay home. Such challenges lead to the fact that interaction between humans and robots for educational purposes needs to be deepened [136], and deepening scientific studies on IoRT technologies will be of the utmost importance in the future.

The growth of the IoRT applications, in addition to the need for developing multi-robot and HRI systems in smart environments, is leading to one of the main issues concerning the era of Industry 4.0: energy consumption. In the past, energy consumption was not a fully addressed concern, as only in the latest years the interest regarding the analysis of energy expenditure has grown among research groups. The difficulty in evaluating and improving the energy efficiency in smart environment is mainly due to the lack of the exact understanding of energy consumption behavior [137]. To overcome this issue, significant attention and efforts must be dedicated to gather information about energy consumption from smart sensors, using different methodologies [138]. For instance, in the context of efficient manufacturing, this information must be integrated into the production management to achieve sustainable processes in the long term.

The processing of large amounts of data in the IoRT applications leads to cyber-security problems. In such systems, it can be necessary to add external calculation and storage capabilities to the IoRT network, as robots, at the edge, may not be able to process and store huge volumes of data. The main cyber-security problems involve insecure communication among users and robots, authentication issues, sensitive data exposing, and weak default robot configuration [139].

As security is fundamental in networked robots, new smart network architectures are becoming essential to protect not only data information, but even humans in HRI-based systems. In fact, safety human–robot collaboration is an issue of critical importance in the industrial environment, mostly when it comes IoRT systems [140], as cyber-attacks through the network or the Internet can compromise the smooth functioning of such industrial systems. To solve such problems, it is fundamental assisting CPS security providers in identifying potential threats by comparing, analyzing and collecting data from several sources. Considering the time expenditure of human and computer to develop IoRT systems, information regarding production and other industrial control applications should be confidential and deeply monitored, as properly collecting the information history could also be a key to assure cyber-physical security. Manufacturing data must remain in the same format, with the same content that it had upon its creation, and must be exchanged with any other suitable industrial system, anywhere in the world, using the same secure protocol.

Cyber-physical security issues can be overcome by using centralized authentication and authorization processes to stream manufacturing information [141], such as data fragments, distributed responsibility, monitored operator control, and granular authorization. As the connection among smart devices is essential to make more and more progress in both industrial and research fields, solving problems related to cyber-physical security is of crucial importance to improve the development of IoRT systems into smart spaces. Therefore, security issues regarding both CPS and robot connection need to be further investigated.

## 5. Conclusions

The presented manuscript outlined those key technologies of Industry 4.0 which led to the development of the IoRT systems. Specifically, an overview of how CPS manages interaction between physical and virtual worlds has been carried out, aiming to introduce the concept of Internet of Robotic Things. The architectures of both systems have been delineated, and different IoRT applications in different smart domains have been outlined, showing how traditional robotic applications have benefited from IoRT-based systems. Specifically, the state-of-the-art from 2018 onwards of the IoRT applications in smart domains has been analyzed, revealing that IoRT systems are now fundamental in different scenarios, starting from the ones included in the industrial field, such as Manufacturing and Agriculture, to the ones that affect the everyday life, such as Health-Care, Education, and Surveillance. IoRT applications can also lay a foundation for the evolution of other domains that are outside the industrial sphere, such as entertainment, visits to museums, or sports competitions, aiming to improve more and more aspects of human life. Moreover, it has been observed that the development of IoRT systems can be the answer to properly deal with the necessity of remote working, where the new requirements of remote interactions between humans and robots could be the answer for more satisfaction and productivity.

This survey has been mainly focused on those IoRT-based systems within the industrial and production fields. IoRT issues and challenges have been deeply analyzed, showing the increasing need for investigation in robot-to-robot cooperation, above all in heterogeneous networks of robots, human–robot interfaces for human-centered interactions, energy management for the optimization of efficiency, and cyber-security to protect sensitive data. The envisaged advancements in efficiency, robustness, and security will open new possibilities in even new domains of applications, which can benefit from the new spread of the latest robotic and network technologies.

## Figures and Tables

**Table 1:** Overview of the literature considered for the Manufacturing domain, from 2018 onwards.

	*Outline of the Works*	*Robot Navigation & Path Planning*	*Data Gathering*	*Image Processing*	*Cloud Computing*	*Multi-Robots*	*HRI*
[50]	Implementation of a vision system on a robot manipulator (ScorBot-ER 9 Pro) to widen the proficiency of the integrated camera-robot system in industrial applications.		✔	✔			
[51]	Automatic path planning of a six-axis robot manipulator for intelligent manufacturing, using a cloud platform that monitors the system through TCP/IP protocol for networked remote controlling and simulation.	✔	✔		✔		
[52]	Integration of robots in CPPS to manage different weight goods, combining UGVs with robot manipulator and air-move systems to built smart factory and smart manufacturing.	✔			✔		
[53]	Cyber physical autonomous mobile robot capable of performing HRI by allowing users to manage orders using a cloud platform. The robot moves following the planned route map, according to the obstacle avoidance system, until it reaches the destination and notifies the cloud platform.	✔			✔		✔
[54]	Systematic development framework called PCDEE-Circle, used for human–robot collaborative disassembly (HRCD) in sustainable manufacturing. A multi-modal perception platform for industrial robots system and human body is defined, by means of a bees algorithm based sequence planning method for an HRCD task.		✔	✔			✔
[55]	Path planning algorithm, using fast marching method (FMM) for a biped robot to move in a static environment, aiming to let it move in both known and unknown scenarios.	✔	✔				
[56]	Mobile robot path planning, combining Cuckoo Search and Bat algorithms to attain the optimal path.	✔	✔				
[57]	Prototype of a system for packing assorted candy, developing a framework to connect consumers, smart factories, and other systems through cloud and logistical networks.				✔	✔	
[58]	Error pattern transformation based on iterative closest point algorithm for object pose estimation of a robot manipulator, using point cloud data gathered from multiple stereo vision systems.	✔	✔	✔			
[59]	Sensorless external force detection produced by human operators in physical HRI, aiming to obtain a dynamic model of an industrial robot manipulator in both dynamic and quasi-static mode.					✔	✔

**Table 2:** Overview of the literature considered for the Agriculture domain, from 2018 onwards.

	*Outline of the Works*	*Robot Navigation & Path Planning*	*Data gathering*	*Image Processing*	*Cloud Computing*	*Multi-Robots*	*HRI*
[78]	Several UAVs are used to collect data by monitoring and mapping the field to vary rate fertilizer, spraying, etc, to reduce crop diseases.	✔	✔	✔		✔	
[77]	Mobile robot equipped with several sensors useful in agriculture (moisture sensor, temperature sensor, contamination sensor, damage of harvest sensor), and controlled by voice recognition, using a smart watch connected to the network.	✔	✔				✔
[79]	Region monitoring of plants in a smart greenhouse, using a cloud-assisted strategy of mobile robots to increase the monitoring region size and reduce time consumption.	✔	✔		✔		
[80]	Remotely configurable crop image acquisition robot system, based on cloud computing and WSN, used to improve the flexibility and adaptation of the mobile robot.		✔	✔	✔		
[81]	Real-time image processing algorithm, using a visual odometry system on a UGV, based on the cross-correlation approach. Low-resolution images are used to attain high accuracy in motion estimation with short computing time.	✔	✔	✔			
[82]	Cooperation among heterogeneous agricultural field robots with a supervisory controller, using a novel approach based on discrete-event system (DES) and the Ramadge-Wonham (RW) theory, which is effective in controlling complex dynamic systems consisting of heterogeneous multi-robot for smart agriculture.	✔	✔			✔	
[83]	Smart agri-system based on embedded electronics, IoT and WSN for agri-farm stock and livestock farms.	✔	✔		✔		
[84]	UGV used for looking for the best suitable deploying position for a WSN system, aiming to analyze the field and gather information about the terrain condition.	✔	✔				
[85]	Automated system developed to control both climate and irrigation in a greenhouse by monitoring temperature, soil moisture, humidity and pH, using a cloud connected mobile robot. Such robot can also discover unhealthy plants using image processing.	✔		✔	✔		
[86]	Deployment of a group of UGVs using a distributed algorithm, aiming to gather data from relevant areas of the field, selected using the Voronoi partitioning.		✔	✔		✔	

**Table 3:** Overview of the literature considered for the Health-Care, Education and Surveillance domains, from 2018 onwards.

	*Outline of the Works*	*Robot Navigation & Path Planning*	*Data Gathering*	*Image Processing*	*Cloud Computing*	*Multi-Robots*	*HRI*
[108]	Cloud and IoT Assisted Indoor Robot (CIoT) for delivery medicine, based on the multi-core embedded system, RFID and IEEE802.11 communication protocol, and cloud platforms.	✔			✔		
[112]	Architecture and design of a wearable affective robot equipped with cognitive computing, named Fitbot. Such robot can perform multi-modal data perception, aiming to recognize the emotions of the patient.		✔		✔		✔
[111]	Generalized IoT-enabled telerobotic architecture designed to support home-centric healthcare system, named Home-TeleBot, realized by integrating human-motion-capture subsystem with robot-control subsystem. The robot used is a dual-arm cooperative robot, named YuMi, which imitates human motion captured by a set of wearable inertial motion capture devices to complete task.	✔	✔				✔
[109]	Realization of a health assessment kiosk, by developing a robotic platform that ensures its functionality within the Smart City information and communication networks, and can provide specific functions by developing applications according to the needs of the patients.	✔					✔
[113]	IoT-based robot system, named InterBot 1.0, equipped with both long-range and short-range communication systems. The robot is efficient in monitoring real-time environments for smart surveillance.	✔	✔				
[114]	Development of a mobile surveillance camera monitoring system, using a line follower to provide a mobile movement, aiming to overcome the limited coverage problem faced by conventional surveillance cameras.	✔		✔			
[115]	Multi-robot system based on swarm intelligence for surveillance and rescue missions, with real-time data uploading on cloud using IoT, exploiting wireless intercommunication between multiple agents, PID technique and ant colony optimization (ACO) algorithm, so that they can accomplish tasks synchronously.	✔	✔	✔		✔	
[116]	Surveillance robot used for climbing both horizontal and vertical surfaces, while automatically controlling surface transitions, exploring space and transmitting live video through wireless channel to the remote workstation.	✔		✔			
[117]	Land mine detection and toxic gas sensing using a multi-purpose field surveillance robot. NodeMCU WiFi is used to interface the controller and do robot, which can climb on any terrains, gathering information. All robotic sensor data are sent to cloud servers.		✔		✔		
[118]	Autonomous Networked Robots (ANR) for surveillance, in which a WSN is implemented, where each sensor node comprises smoke, infrared fire, odor, and motion detector sensors, and RF transceivers for networking and communication.	✔	✔			✔	
